# Preliminary Medical Education

**Published:** 1902-12-13

**Authors:** 


// The Hospital.

A Journal of

The Medical Sciences and Hospital Administration.

Vol. XXXIII.—No. 846.] Edited by Sir Henry Burdett, K.C.B., and Solomon C. Smith, M.D., M.R.C.P. [December 13, 1902.

### PRELIMINARY MEDICAL EDUCATION.

The probability to which we referred last week, that in the near future an attempt will be made to establish complete reciprocity, as regards medical education and practice, among the constituent states of which the British Empire is composed, confers increased importance upon the whole question of the conditions, as regards preliminary training, under which that education is likely to be conducted. To the solution of this question the week has afforded two contributions worthy of notice: in the reply given by the Education Committee of the General Medical Council to the President and Council of the British Medical Association, and in the publication of an address delivered by Sir Dyce Duckworth at the opening in October of the winter session of the medical department of Owens College. The British Medical Association among other things, recommended that no one should be registered as a medical student under the age of 17, that the standard of examination in preliminary general education required by the Council as a condition of registration should be suitable for candidates of the stated age, that there should be a Pass and an Honours division, and that the Medical Acts should be so amended as to enable the Council to appoint a single preliminary Examining Board for the purpose of securing uniformity. The Education Committee had to point out that the requirement of registration was made by the Council without authority as a matter of convenience; that the Council is unable to enforce it, or to refuse admission to the Medical Register to any qualified persons who have never been registered as students at all, but who, nevertheless, have been admitted to examination by qualifying bodies which, in this respect, are recalcitrant with regard to the wishes of the Council; and that there is not the slightest probability that any Government would introduce, or any English Parliament enact, provisions which would be strenuously opposed by every university and institution for higher teaching in the United Kingdom, which would be out of harmony with the educational arrangements of every other profession, and which would inevitably tend to reduce the preliminary education of all medical students to the minimum which alone could be enforced by any general regulation. We apprehend that, from a practical point of view, these considerations may be taken to be conclusive; but they nevertheless entirely fail to touch the principles by which, in so far as possible, a national or an imperial policy should be controlled.

There is, we believe, an assured conviction, among all who are old enough to possess practical knowledge of the subject, that the preliminary education of medical students to-day is inferior to that of fifty years ago. They have often been taught more, but they seldom know so much; and we regard this as the explanation of a statement made by Sir Dyce Duckworth, who said: "No one who considers the matter with an open mind can believe that the medical profession holds its legitimate place in public estimation. Be the reasons what they may, I believe that estimate to be lower now than was the case half a century ago." The preliminary education of to-day as it is tested by the prevailing examinations, seems to us to be to a large extent illusory in its character, and to be much concerned in imparting, again to quote Sir Dyce, not so much knowledge which is really acquired, as "that counterfeit of it which is merely hired for the occasion." Hence "the bachelors of arts who write and speak their own language incorrectly, and are unable to construe a simple Latin sentence." Hence, again, "the mental confusion and delay constantly experienced by an unintelligent appreciation of terms derived from Latin and Greek," and hence, may we not add, the mental state of that practitioner who composed the word "*dacryocystosyringocatacleisis*" in order to express a closure of the tear-duct. However this may be, the Education Committee of the Council is not without fear that the tests now employed are more severe than they should be, and that they exclude large numbers of young men from the medical profession. If it be to the advantage of the public that the supply of medical men should be maintained by relaxing the requirements of study and examination, we shall not be far from the conclusion at which we understand Sir Dyce Duckworth to have arrived, namely, that it is expedient to have a general recognition of two standards of qualification—that which implies a fitness for the ordinary demands of practice, and that which implies the possession of distinctly higher attainments. Something of this kind is already afforded, in surgery, by the respective diplomas of Fellow and Member of the English College; and a similar scheme might possibly be extended to other departments of the healing art. Such a plan might afford a distinction between the mere practitioner and the man of science, but it would have the drawback that the latter, although as "chock-full of science" as Solomon Gills himself, might nevertheless be deficient in the powers of observation and in the clinical instincts which are the most valuable of all possessions in the sick-room.

